# Adherence to the British Orthopaedic Association Standards for Trauma (BOAST) Guidelines for the Management of Neck of Femur Fractures

**DOI:** 10.7759/cureus.117832

**Published:** 2026-10-13

**Authors:** Ehizokhale O Okhibhamen, Ali Almahfoodh, Michael Osumah, Khalid M Sohaib, Roshan Jacob, Abidemi M Adisa, Mahwish Naureen

**Affiliations:** 1 Department of Trauma and Orthopaedics, Manchester University NHS Foundation Trust, Manchester, GBR; 2 Department of Trauma and Orthopaedics, Salford Royal NHS Foundation Trust, Manchester, GBR

**Keywords:** boast guidelines, compliance, femoral neck, hip fracture, management

## Abstract

Introduction

Over 72,000 hip fractures occur annually in the United Kingdom, costing over £2 billion, with 10% mortality at 30 days and 30% annually. Therefore, the study aimed to compare hip fracture care at North Manchester General Hospital with the British Orthopaedic Association Standards for Trauma (BOAST) guidelines.

Methodology

A retrospective review of electronic patient records at Manchester University NHS Foundation Trust was conducted from September to November 2025. Patients aged >60 years with native intracapsular or extracapsular femoral neck fractures, or those aged >60 years with pathological femoral neck fractures, were included. Descriptive analysis was performed to ascertain compliance with BOAST guidelines.

Results

The management of 51 patients was audited against 13 BOAST parameters. 100% compliance was achieved in seven parameters: certainty of diagnosis, a multidisciplinary team (MDT) approach to patient care, preoperative optimisation by an orthogeriatric team within 72 hours, documented weight-bearing status, falls risk assessment, bone health assessment, and data entry into the National Hip Fracture Database (NHFD). Parameters with less than 100% compliance were post-operative physiotherapy (50, 98%), venous thromboembolism (VTE) prophylaxis (50, 98%), analgesia (46, 91%), VTE assessment (45, 88%), post-operative delirium assessment (43, 84%), and surgery within 36 hours (32, 62.8%). Reasons for non-compliance included transfer to the intensive care unit (ICU) for post-operative physiotherapy; workload-related delays in VTE prophylaxis; unfamiliarity with local analgesia protocols among newly appointed National Health Service (NHS) doctors; inconsistent documentation of VTE assessment; and inconsistent completion of post-operative delirium assessments, with unclear responsibilities between doctors and coordinators. Limited theatre capacity and the need for medical optimisation accounted for non-compliance with the 36-hour surgery target.

Conclusions

BOAST compliance was good overall, but gaps in documentation, timing, and role clarity remain. Improvements include enforcing admission checklists, recording arousal, attention, and acute change using the abbreviated mental test-4 (4AT), pharmacist incident reporting, trauma coordinator oversight, and neck of femur training.

## Introduction

Hip fracture is one of the most common orthopaedic emergencies affecting older people worldwide [1]. As the elderly population ages, maintaining functionality becomes increasingly challenging, making independence and improved mobility the main objectives following therapeutic intervention for hip fractures [2]. These fractures are associated with increased mortality, high morbidity rates, permanent disability, socioeconomic costs, and psychological issues such as depression [3,4]. By 2050, the number of hip fractures worldwide is predicted to exceed 4.5 million [4]. Annually, approximately 72,000 people in the United Kingdom sustain a hip fracture, accounting for one in 30 hospital bed spaces being occupied and an economic burden of around £2 billion [1].

Neck of femur (NOF) fractures are among the major types of hip fractures affecting older people and are more prevalent in white women than in men [4]. They are estimated to account for 60% of hip fractures in patients aged 60-69 years [5]. The incidence of NOF fractures is directly related to ageing, with the risk of fracture doubling every decade among people aged 50 years and above [6]. These fractures are commonly caused by low-impact falls in older people, whereas in younger people, they are associated with high-impact falls and vehicular accidents [7]. The annual mortality rate following NOF fractures has been reported to be as high as 30% [8].

Age is a major risk factor for hip fractures due to the increased rates of osteoporosis and fall-related injuries among the elderly population [3]. Gender and race have also been associated with hip fractures, with women being at greater risk than men. At the same time, White and Asian populations have been reported to have higher rates of hip fractures than Black populations [4]. Other risk factors for hip fractures include falls, osteoporosis, chronic medication use, and reduced activity levels [6]. The presence of comorbidities, particularly in the elderly population, increases the risk of complications in patients with NOF fractures, potentially resulting in long-term care requirements, functional decline, and mortality [6]. Similarly, gender is associated with outcomes following NOF fractures, with men having lower mortality rates than women [9].

The care of older or frail patients with orthopaedic trauma depends on the effectiveness of the multidisciplinary team (MDT) in managing all aspects of the patient's health, including physical injury treatment, comorbidity management, rehabilitation, and injury prevention [10]. The British Orthopaedic Association Standards for Trauma (BOAST) guidelines provide evidence-based recommendations for injury management and rehabilitation, aiming to reduce mortality among older or severely ill orthopaedic patients [11]. The BOAST guidelines emphasise prompt admission, surgery within 36 hours of presentation, orthogeriatric assessment, effective analgesia, venous thromboembolism prophylaxis, nutritional assessment, early mobilisation, falls assessment, secondary prevention of osteoporosis, and data entry into the National Hip Fracture Database (NHFD), Fracture Liaison Service Database (FLS-DB), and Trauma Audit and Research Network (TARN) [10,12].

Adherence to standard protocols, such as the BOAST guidelines and other international guidelines, where applicable, has been associated with decreased mortality, improved functional recovery, lower complication rates, and shorter hospital stays [1,13-15]. Therefore, this study aimed to compare hip fracture care at North Manchester General Hospital with BOAST standards and identify the underlying reasons for any areas of non-compliance.

## Materials and methods

Study design and setting

This was a retrospective clinical audit conducted at North Manchester General Hospital, part of Manchester University NHS Foundation Trust, United Kingdom.

Study duration

The audit was conducted over three months, from 1 September to 30 November 2025.

Sampling technique

Purposive sampling was done to easily review the records of patients from the electronic patient records (HIVE) and select patients based on the study criteria.

Sample size determination

The sample size for the study was determined using two formulas.

Uncorrected Sample Size (Cochran's Formula)

The initial sample size was calculated using Cochran's formula, where *N*_0_ represents the uncorrected sample size, *Z* is the standard normal deviate corresponding to a 95% confidence level (1.96), *p* is the estimated proportion of patients with neck of femur fractures (0.5 or 50%), and *d* is the desired precision level (0.10 or 10%):

$N_0 = \frac{Z^2 p(1-p)}{d^2} = \frac{(1.96)^2(0.5)(1-0.5)}{(0.10)^2} = 96.04$

Finite Population Correction (FPC)

FPC was applied because of the limited number of eligible patients in the study population. This adjustment was used to refine the initial sample size calculated using Cochran's formula. Here, *N*_0 _is the uncorrected sample size, and *N* represents the total estimated population of eligible patients during the audit period (100):

$n_{\mathrm{adj}} = \frac{N_0}{1+\frac{N_0-1}{N}} = \frac{96.04}{1+\frac{96.04-1}{100}} = \frac{96.04}{1.9504} \approx 49.24$

The adjusted sample size was approximately 50 eligible patients.

Patient selection criteria

Only patients who met the inclusion criteria were recruited into the study. The inclusion criteria were patients aged >60 years with native intracapsular or extracapsular femoral neck fractures, including those with metabolic bone diseases, and patients aged >60 years with pathological femoral neck fractures. The exclusion criteria were patients with periprosthetic femoral fractures and those expected to die within two to three days.

Data collection

A review of the electronic patient records (HIVE) at North Manchester General Hospital was conducted to extract information on eligible patients.

Study parameters

The variables collected included demographics, documentation quality, preoperative care, surgical intervention, and various post-operative management activities.

Audit standard

The BOAST guidelines for the management of hip fractures were used. The criteria against which hip fracture care was assessed were as follows: certainty of diagnosis or, if uncertain, advanced scanning following the Post-Take Ward Round (PTWR); analgesic prescriptions tailored to patient needs; venous thromboembolism (VTE) prophylaxis; VTE prophylaxis prescribed on admission; surgery within 36 hours of diagnosis; an MDT approach to patient care (anaesthetics, orthopaedics, nursing, and orthogeriatrics); preoperative optimisation by the orthogeriatric team within 72 hours; weight-bearing status documented in the operative note; post-operative delirium assessment; physiotherapy assessment on the first day after surgery; falls risk assessment during admission; bone health assessment during fracture care; and data entry into the NHFD.

Data analysis

Descriptive analysis using frequencies and percentages was performed to measure compliance with the BOAST standards.

Determination of non-compliance

The reasons for non-compliance were determined using the documentation provided in each patient's clinical records.

Ethical approval

Ethical approval was not required for this clinical audit, as Manchester University NHS Foundation Trust does not issue ethical approval for internal clinical audits. Instead, the hospital registers the audit and provides a unique code. This study was registered under audit code Ulysses ID 12476. 

## Results

A total of 51 patients met the study criteria, and their hip fracture care and management were assessed against 13 BOAST standards.

Table 1 demonstrates a generally high level of adherence to BOAST during the pre-operative management of patients with neck of femur fractures, reflecting strong compliance with recommended evidence-based practices. The highest compliance was observed in diagnostic confirmation, MDT involvement, and timely orthogeriatric review, indicating that patients consistently received comprehensive assessment and optimisation before surgery. These findings suggest the presence of well-established clinical pathways that facilitate collaborative decision-making and holistic patient care. Similarly, preventive measures, including VTE prophylaxis and individualised pain management, showed excellent compliance, although formal VTE risk assessment demonstrated slightly lower adherence, implying that documentation processes may require further strengthening despite appropriate clinical practice.

**Table 1:** Pre-operative compliance assessments against BOAST guidelines. PTWR, Post-Take Ward Round; VTE, venous thromboembolism; MDT, multidisciplinary team; BOAST, British Orthopaedic Association Standards for Trauma

Standards	Achieved compliance (%)	Patient frequency (*n*) for achieved compliance	Expected compliance (%)
Certainty on diagnosis/if uncertain on PTWR, proceed with advanced scanning	100	51	100
Analgesic prescriptions tailored to patient needs	91	46	100
VTE assessment	88	45	100
VTE prophylaxis	98	50	100
Surgery done within 36 hours of diagnosis	62.8	32	100
MDT approach to patient care	100	51	100
Pre-operative/optimisation by orthogeriatric within 72 hours	100	51	100

In comparison with other pre-operative parameters, the timing of surgical intervention showed the lowest level of compliance, highlighting it as the most significant deviation from BOAST recommendations. This finding suggests that organisational or system-related factors, such as theatre availability, perioperative optimisation, or workforce limitations, may contribute to delays despite adequate patient preparation. Overall, the comparison of study parameters indicates that standards dependent on clinical assessment and multidisciplinary collaboration were consistently achieved, whereas those influenced by institutional logistics were comparatively more difficult to meet, identifying timely surgery as the primary area requiring quality improvement.

Table 2 indicates excellent adherence to BOAST recommendations during the post-operative management of neck of femur fractures, demonstrating the effectiveness of established post-operative care pathways within the institution. The majority of assessed standards, including documentation of weight-bearing status, falls risk assessment, bone health assessment, and participation in the NHFD, were consistently achieved, reflecting a comprehensive approach to rehabilitation, secondary fracture prevention, and clinical governance. Early physiotherapy assessment also demonstrated very high compliance, emphasising the integration of prompt rehabilitation into routine post-operative care to support functional recovery and reduce complications associated with prolonged immobilisation.

**Table 2:** Post-operative compliance assessments against BOAST guidelines. BOAST, British Orthopaedic Association Standards for Trauma

Standards	Achieved compliance (%)	Patient frequency (*n*) for achieved compliance	Expected compliance (%)
Weight-bearing status documented on operative note	100	51	100
Delirium assessment done post-operatively	84	43	100
Physiotherapy assessment day 1 after surgery	98	50	100
Falls risk assessment	100	51	100
Bone health assessment	100	51	100
Data entry into the National Hip Fracture Database (NHFD)	100	51	100

Compared with the other post-operative parameters, delirium assessment showed relatively lower compliance, making it the principal area requiring improvement. Given the high incidence and clinical significance of post-operative delirium among elderly patients with hip fractures, inconsistent assessment may delay early recognition and management, potentially affecting recovery and length of hospital stay.

Overall, comparison of the study parameters reveals that standardised protocol-driven interventions achieved more consistent adherence than assessments requiring ongoing clinical observation and documentation. These findings suggest that while post-operative care is generally delivered in accordance with national BOAST standards, greater emphasis on routine delirium screening would further strengthen compliance, enhance patient safety, and improve the quality of post-operative outcomes for patients undergoing hip fracture surgery.

Figure 1 illustrates that the reasons for non-compliance varied across the assessed BOAST standards, with delays related to theatre capacity and patient medical optimisation emerging as the most prominent barrier. These reasons for non-compliance were obtained from the documented information in the clinical records of the patients. In comparison, deficiencies associated with delirium assessment and unfamiliarity with local analgesia protocols occurred less frequently but remained important contributors to non-adherence. Documentation gaps, including unclear reasons for omitted VTE assessment, were more common than workload-related delays affecting VTE prophylaxis and post-operative intensive care admission influencing physiotherapy assessment. Overall, system-level constraints contributed more substantially to non-compliance than individual clinical practice, highlighting opportunities to improve service organisation, documentation, and staff education.

**Figure 1:**
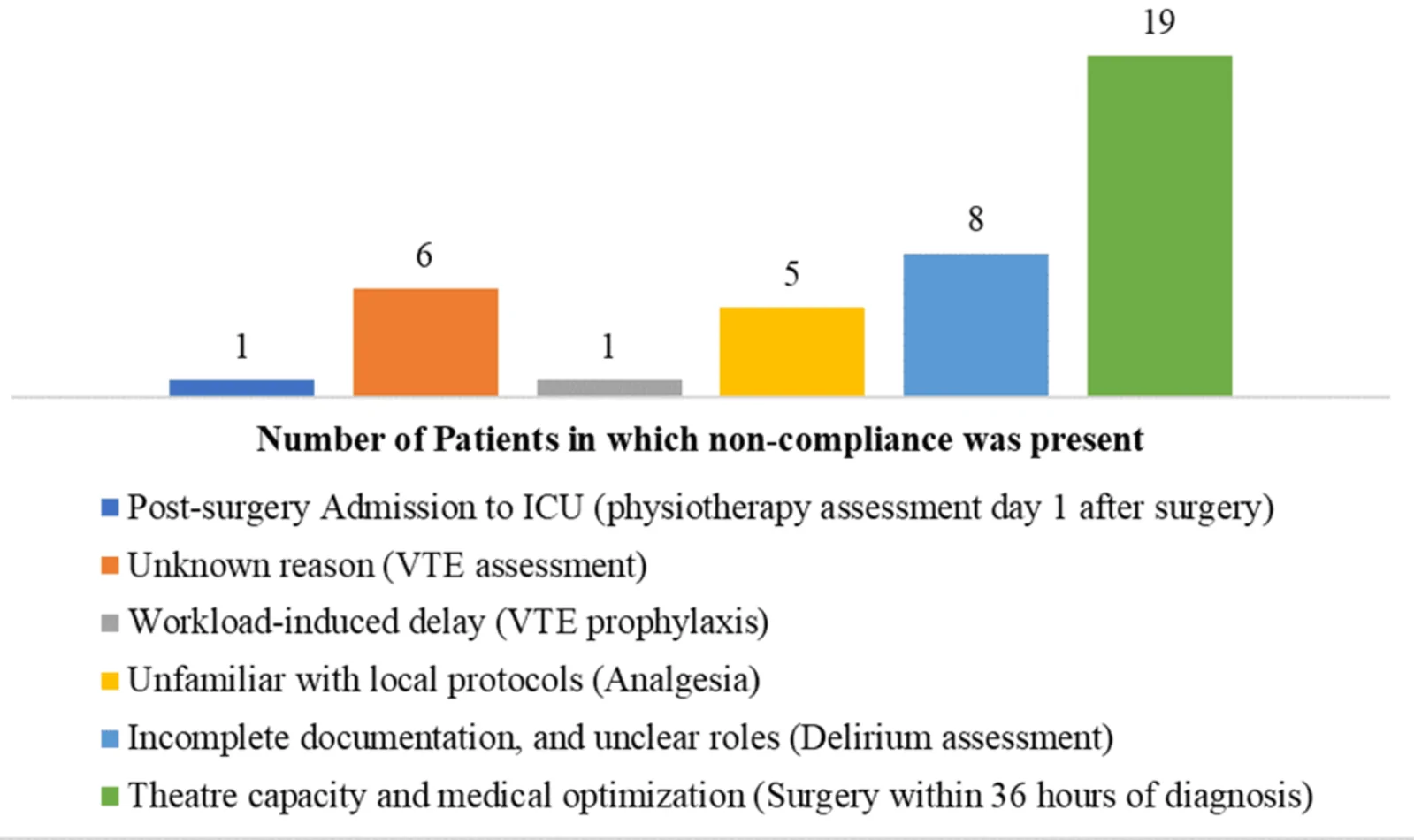
Documented reasons for non-compliance with the six BOAST standards. BOAST, British Orthopaedic Association Standards for Trauma; VTE, venous thromboembolism

## Discussion

The audit showed relatively good compliance with the BOAST standards, as seven of the 13 assessed standards had 100% compliance. These included certainty of diagnosis, an MDT approach to patient care, pre-operative optimisation by the orthogeriatric team within 72 hours, documented weight-bearing status, falls risk assessment, bone health assessment, and data entry into the NHFD.

The immediate admission of elderly patients with hip fractures to a dedicated orthogeriatric ward has been shown to improve patients’ mobility outcomes at four months. The 100% compliance with pre-operative optimisation by the orthogeriatric team within 72 hours was higher than the 82% and 52.9% reported in previous studies [16,17], respectively. Another study showed that the use of an MDT approach to patients with NOF fractures helped reduce the risk of delirium and complications such as bleeding, cardiac issues, thromboembolism, and infections [6].

The reasons for non-compliance were investigated and identified using documentation obtained from patients’ clinical records. For the physiotherapy assessment on day one after surgery, compliance was 50 (98%). The reason for non-compliance was that one patient had to be admitted to the intensive care unit (ICU) post-operatively; hence, the patient could not start physiotherapy. This could be considered an “unavoidable” reason, as the patient remained in a critical condition after surgery. For VTE prophylaxis, compliance was 50 (98%), and non-compliance was due to a workload-induced delay.

Prescribed analgesia should be specific to the pain threshold and must incorporate the needs of patients with hepatic and renal diseases [11]. Although our study showed 46 (91%) compliance with analgesic prescriptions, non-compliance among five patients was mainly attributed to newly joined National Health Service (NHS) doctors who were unfamiliar with local protocols. Before any surgical intervention, VTE assessment for each patient must be carried out, and the associated risk must be clearly documented [11]. However, our study showed only 45 (88%) compliance with this standard. The reasons for non-compliance with VTE assessment were unclear, as six patients had no documented VTE assessment and the reasons were unknown.

Post-operative delirium assessment had only 43 (84%) compliance. The reasons for non-compliance were twofold: incomplete and inconsistent documentation, and unclear roles between doctors and coordinators. The 84% compliance in our study was higher than the 80% reported in a previous study [16]. Each healthcare facility is advised to have a delirium policy that outlines preventative measures, individualised interventions, and the prompt identification of potentially reversible issues [11].

Surgery within 36 hours of diagnosis had the lowest compliance, with 32 (62.8%) of the 51 patients undergoing surgery within the designated time. The importance of urgent surgery in patients with NOF fractures is to reduce the risk of perioperative complications, increase patient comfort, and ultimately reduce the length of hospital stay [18].

Comparatively, compliance in our study was lower than the 65% and 73.5% reported in similar audits [16,19], respectively. However, it was considerably higher than the 25% reported by Ormiston et al. [20] in their audit of the management of fragility femoral fractures against BOAST guidelines. This disparity could be attributed to the fact that the study by Ormiston et al. [20] was a multicentre audit that included several types of fragility femoral fractures, whereas our study was a single-centre study that assessed only NOF fractures.

The major reasons for non-compliance with the requirement for surgery within 36 hours of diagnosis in our study fell into two categories: 12 (23.5%) patients experienced delays due to theatre capacity, while 7 (13.7%) required medical optimisation, resulting in delays for these 19 patients. Limited theatre capacity was also identified as the leading cause of surgical delays, with over 60% of cases attributed to this factor in an audit conducted by Jamal et al. [21].

Limitations of the study

The authors recognise the small sample size assessed within the study timeframe as a weakness, as it did not allow the inclusion of other potentially eligible participants. First, this was a single-centre study that audited only North Manchester General Hospital, without the participation of other hospitals within Manchester University NHS Foundation Trust. Hence, the study may lack generalisability compared with clinical audits involving all hospitals within a specific NHS trust. Second, reliance on documented observations to determine the reasons for non-compliance with some BOAST standards is a limitation, as no inferential statistical analyses were conducted to determine the significance of or relationships between variables affecting compliance with the audited standards. Third, the non-inclusion of baseline patient characteristics and frailty assessment is another limitation.

## Conclusions

Compliance with BOAST guidelines at North Manchester General Hospital was good overall, although important gaps in documentation, timing, and role clarity remain. Therefore, areas for improvement include enforcing admission checklists, recording the arousal, attention, and acute change components of the abbreviated 4AT, pharmacist incident reporting, trauma coordinator oversight, and induction training for NOF fractures.
